# Cardiorespiratory Fitness is Inversely Associated with Risk of Low Bone Mineral Density in Older Korean Men

**DOI:** 10.3390/ijerph17217907

**Published:** 2020-10-28

**Authors:** Inhwan Lee, Jeonghyeon Kim, Hyunsik Kang

**Affiliations:** College of Sport Science, Sungkyunkwan University, Suwon 16419, Korea; ansh00@naver.com (I.L.); zzagkim115@naver.com (J.K.)

**Keywords:** physical activity, cardiorespiratory fitness, bone health, older adults

## Abstract

Little is known regarding the association between physical fitness and bone health in older Korean men. This study investigated the relationship between estimated cardiorespiratory fitness (eCRF) and bone mineral density (BMD). This cross-sectional study included 2715 Korean men aged 50 years and older selected from those who participated in the 2008–2011 Korea National Health and Nutritional Examination and Survey. eCRF was obtained using a sex-specific algorithm developed on the basis of age, body mass index, resting heart rate, and physical activity and classified into low, middle, and high categories. Femoral neck BMD was assessed by dual X-ray absorptiometry. Odds ratios (OR) and 95% confidence intervals (CI) for osteopenia, osteoporosis, and low BMD were calculated for eCRF categories in models fully adjusted for age, waist circumference, education, income, smoking, heavy alcohol intake, serum vitamin D, serum parathyroid hormone, and dietary intake of energy, protein, calcium, and vitamins A and C. Overall, eCRF levels were positively associated with BMD and negatively with prevalence of osteopenia, osteoporosis, and low BMD. Logistic regression showed inverse trends in the risks of osteopenia (high vs. low: OR = 0.692; 95% CI, 0.328–0.517; *p* = 0.049) and low BMD (high vs. low: OR = 0.669; 95% CI, 0.497–0.966; *p* = 0.029) by eCRF category in models fully adjusted for all the measured covariates. The current findings suggest that maintaining high eCRF via regular physical activity may contribute to attenuation of age-related loss of BMD and decreased risk for low BMD in older Korean men.

## 1. Introduction

Osteoporosis is a skeletal disease characterized by low bone mass and deterioration of bone structural integrity, leading to bone fragility and increasing the risk for bone fracture. Aging is an established risk factor of the disease, and its prevalence is predominantly in adults aged 50 years and older [1]. With a rapidly rising elderly population, osteoporosis has become a serious public health issue in both developed and developing countries [2]. The prevalence of osteoporosis is also associated with behavioral risk factors such as smoking, heavy alcohol consumption [3], dietary intake [4,5], vitamin D deficiency [6], and physical inactivity [7] in conjunction with low socio-economic status [8].

In South Korea, low bone mineral density (BMD) in older men is an underappreciated health issue. Older Korean men have a lower prevalence of osteoporosis and a lower standardized prevalence of vertebral fractures using the age distribution of the Korean population than older Korean women [9,10,11]. Interestingly enough, however, older Korean men are at higher risk of post-hip-fracture mortality compared with older Korean women [12,13], with the highest standardized mortality rates seen in the age group of 50–59 years [12]. By analyzing nationwide data involving Korean populations aged 50 years and older, Ha [14] reported that the prevalence of osteoporosis ranged from 6.1% to 13.1% in men and from 24.3% to 35.5% in women. The incidence rate of osteoporotic fracture was lower in men than in women (i.e., 110.5/100,000 in men vs. 243.1/100,000 women. However, men had approximately 1.4- and 2.2-fold higher risks of post-spinal-fracture mortality [15] (Kim et al., 2016) and post-hip-fracture mortality [16], respectively, compared with women. Given the relationship between osteoporosis and mortality, therefore, maintaining BMD is vital to men’s bone health later in life.

Both resistance exercise and weight-bearing aerobic exercise are recommended as a primary therapeutic strategy for the prevention and treatment of low BMD, including osteopenia and osteoporosis [17]. Observational studies showed that physically active individuals had attenuated age-related decline in BMD compared with physically inactive ones [18]. In addition to physical activity, recent studies examined the relationship between cardiorespiratory fitness (CRF) and BMD in older adults and reported an attenuating effect of high CRF on age-related declines in BMD in older women [19] and men [20]. To the best of our knowledge, however, little is known regarding the role of CRF as a prognostic tool in determining the risk of osteopenia and osteoporosis in older Korean adults. Therefore, an assessment of the relationship between CRF and BMD will provide the opportunity to develop better therapeutic strategies for Korean patients with osteoporosis.

CRF reflects the maximal capacity of the respiratory and cardiovascular systems to supply oxygen to working skeletal muscles during exercise and represent an objective indicator of habitual physical activity. In older populations, however, there is limited evidence demonstrating the prognostic role of CRF in relation to bone health status. An objective assessment of CRF requires a direct measurement of oxygen consumption with gas analyzers and well-trained personnel during a graded exercise test, which is expensive, time-consuming, and often difficult to implement in a population-based study. Alternatively, age-sex-specific algorithms consisting of routinely obtained health measures have been developed to estimate CRF (eCRF) with acceptable accuracy [21]. Therefore, this study aimed to investigate the relationship between eCRF and BMD in a cohort of Korean men aged 50 years and older selected from the participants of the 2008–2011 Korea National Health and Nutritional Examination and Survey (KNHANES) IV and V.

## 2. Materials and Methods

### 2.1. Data Source and Study Participants

In this study, we focused only on men aged 50 years and older (*n* = 5872) from the 2008–2011 KNHANES IV and V, nationwide surveys designed to assess health and nutritional status of the Korean population. A detailed description of the KNHANES including the sampling design is available elsewhere [22].

**Figure 1:**
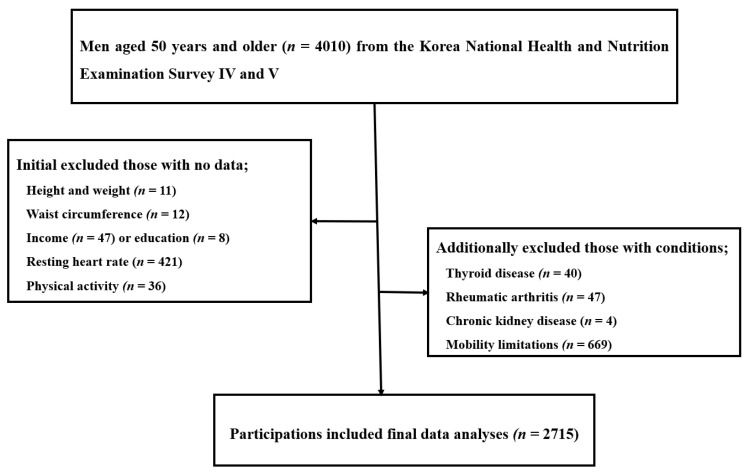
Selection of eligible study participants.

We initially selected men aged 50 years and older (*n* = 4,010) who underwent dual-energy X-ray absorptiometry (DEXA)-based assessment of bone mineral density. We excluded women or men who had no data available regarding height and weight (*n* = 11), waist circumference (*n* = 12), income (*n* = 47), education (*n* = 8), physical activity (*n* = 36), or resting heart rate (RHR) (*n* = 421). Additionally, men with thyroid disease (*n* = 40), rheumatic arthritis (*n* = 47), mobility limitations (defined as any orthopedic or physical impairment and/or an inability to move) (*n* = 669), or chronic kidney diseases (*n* = 4) were also excluded. Consequently, a total of 2715 men were included in final data analyses (Figure 1). The Korea Centers for Disease Control and Prevention institutional review board reviewed and approved the 2008–2011 KNHANES IV and V surveys (2008-04EXP-01-C, 2009-01CON-03-2C, 2010-02CON-21-C, 2011-02CON-06-C) in accordance with the Declaration of Helsinki. Informed consent form was obtained from all the participants.

### 2.2. Study Variables

#### 2.2.1. Assessment of Bone Mineral Density

BMD was measured at the femoral neck by DEXA with the Hologic Discovery QDR4500W bone density unit (Hologic Inc., Bedford, MA, USA). Quality control and calibration standards were conducted as specified by the manufacturer, with a precision coefficient of variation of 2.5%. Osteoporosis was defined as a T-score of −2.5 or less, and osteopenia was defined as a T-score of −1.0 or less and greater than −2.5. For regression modeling, T-score thresholds of −1.0 or less and greater than −2.5 (osteopenia), −2.5 or less (osteoporosis), and −1.0 or less (low BMD) were utilized. The reference values for BMD were determined from a reference group of young healthy adults aged 20–29 years in accordance with the recommendations of the World Health Organization (WHO) [9].

#### 2.2.2. Estimation of Cardiorespiratory Fitness

Estimation of CRF (eCRF) was obtained in units of metabolic equivalents (METs) by using a sex-specific algorithm as previously reported [21]; eCRF (METs) = 2.77 (sex) − 0.10 (age) − 0.17 (body mass index) − 0.03 (resting heart rate) + 1.00 (physical activity score) + 18.07. Physical activity score was assessed according to the Johnson Space Centre (JSC) Physical Activity Rating (PAR) scale [23] in the range of 0 (inactive) to 7 (highly active with more than 3 h per week). eCRF was classified into low (lowest 25%), middle (middle 50%), and high (highest 25%).

#### 2.2.3. Determination of Covariates

Height (m) and weight (kg) were measured using a digital mobile stadiometer (seca 274, SECA, Hamburg, Germany) and a portable bench scale (GL-6000-20, G-tech, Seoul, Republic of Korea), respectively, with light clothing and no shoes. Body mass index (BMI) was calculated in unit of weight (kg)/height (m^2^). Waist circumference (WC in unit of cm) was measured at the narrowest location between the iliac crest and the lower border of the rib cage using a retractable measuring tape (seca 201, SECA, Hamburg, Germany). Parameters of socio-economic status and health behaviors, such as education (i.e., elementary, middle, and high school, and college and higher), household income, smoking (past and current smokers), and heavy drinking (seven or more standard drinks with a frequency of two times or more per month), were assessed with the health interview questionnaire described previously [22]. Blood samples were collected from antecubital veins after an overnight fast to assess serum levels of 25-hydroxyvitamin D3 (25(OH)D) and parathyroid hormone (PTH) by radioimmunoassay (1470 Wizard gamma counter; PerkinElmer, Turku, Finland) and the N-tact PTH assay with LIAISON (Diasorin^®^ Analyzer, Centralino, Italy), respectively. A face-to-face interview by a well-trained nutritionist was performed to assess dietary intakes of daily total energy, protein, calcium, and vitamin A and C supplements. A detailed description of the health and nutritional examinations and blood chemistry profiles is available elsewhere [22].

### 2.3. Statistical Analyses

All statistics of this survey have been calculated using sample weights assigned to sample participants [22]. Data are presented as mean ± standard deviation (SD) and percentage (%) for continuous and categorical variables, respectively. The Cochran–Mantel–Haenszel test and linear regression were used to test any significant trends in categorical and continuous variables, respectively, according to eCRF category (from low to high). Logistic regression was used to calculate odds ratio (ORs) and 95% confidence intervals (CIs) for osteopenia, osteoporosis, and low BMD according to eCRF category (from low to high). Statistical significance was tested at *p* = 0.05. All statistical analyses were conducted with the SPSS-PC statistical software (version 23.0, SPSS, IBM, Armonk, NY, USA).

## 3. Results

Table 1 represents the characteristics of the study participants by eCRF category. Negative linear trends were found in age (*p* < 0.001), BMI (*p* < 0.001), WC (*p* < 0.001), RHR (*p* < 0.001), and heavy alcohol consumption (*p* < 0.001) by eCRF category (from low to high). The middle and high eCRF groups were younger and had lower means of BMI, WC, RHR, and heavy alcohol intake compared with the low eCRF group. On the other hand, the middle and high eCRF groups were more active (*p* < 0.001) and had higher means of smoking (*p* < 0.001) and education (*p* < 0.001) compared with the low eCRF group.

With respect to blood chemistry and nutrients, positive linear trends were observed in vitamin D (*p* = 0.004), energy intake (*p* < 0.001), protein (*p* < 0.001), calcium (*p* < 0.001), vitamin A (*p* < 0.001), and vitamin C (*p* < 0.001) by eCRF category. The middle and high eCRF category groups had higher values of serum vitamin D, caloric intake, and daily intakes of protein, calcium, vitamin A, and vitamin D, compared with the low eCRF category group. On the other hand, an inverse linear trend was observed in serum PTH (*p* < 0.001) by eCRF category. The middle and high eCRF category groups had a lower level of serum PHT compared with the low eCRF category group.

Table 2 describes BMDs and T-scores of the femoral neck by eCRF category. Positive linear trends were found in BMD (*p* < 0.001) and T-score (*p* < 0.001) by eCRF category (from low to high). The middle and high eCRF category groups had higher BMD and T-scores compared with the low eCRF category group. In addition, inverse linear trends were observed in the prevalence of osteopenia (*p* < 0.001) and osteoporosis (*p* < 0.001) by eCRF category. The middle and high eCRF category groups had a lower prevalence of osteopenia and osteoporosis compared with the low eCRF category group.

Table 3 represents the odds ratios for osteopenia, osteoporosis, and low BMD by eCRF category (from low to high). The middle and high eCRF groups had ORs of 0.647 (*p* < 0.001) and 0.412 (*p* < 0.001) for osteopenia, respectively compared with the low eCRF group (OR = 1). The OR for the high category group remained statistically significant (*p* = 0.049) even after adjustments for all the measured covariates. In addition, the middle and high eCRF groups had ORs of 0.241 (*p* < 0.001) and 0.080 (*p* < 0.001) for osteoporosis, respectively compared with the low eCRF group. The ORs for the middle and high eCRF groups were no longer significant after adjustments for all the measured covariates. Finally, the middle and high eCRF groups had 40% (*p* < 0.001) and 63% (*p* < 0.001) lower risks, respectively, of low BMD compared with the low eCRF group. The OR for the high eCRF group remained significant (*p* = 0.029) even after adjustments for all the measured covariates.

## 4. Discussion

In this study, we examined the relationship between eCRF and femoral neck BMD in a sample of 2715 Korean men extracted from those who participated in the 2008–2011 KHHANES IV and V. We are the first to report that eCRF is significantly and inversely associated with risks of osteopenia and osteoporosis in otherwise healthy older Korean men. In addition, the findings of this study showed that low BMD at the femoral neck is a widespread problem in older Korean men with a prevalence of approximately 41%.

The findings of the study agreed with previous studies that reported the association between higher CRF and better bone health in older adults. For example, Wainstein et al. [20] examined the association between objectively measured CRF and BMD at the femoral neck in 2569 men aged 50–90 years and found that higher BMD was significantly associated with lower risks for osteoporosis and low BMD. Schwarz et al. [24] examined the relationships of muscular strength and CRF with BMD at the total hip and lumbar spine in 153 Danish men aged 31 to 60 years. Objectively measured CRF was positively associated with the T-scores of the total hip and lumbar spine in models fully adjusted for age, weight, height, smoking, alcohol intake, and leisure time physical activity. The positive association between CRF and BMD was also reported from a cohort of community-dwelling 802 elderly Portuguese men and women [25]. Together, these findings suggest that having or maintaining high CRF is significantly associated with attenuated age-related loss in BMD and a lower risk of osteopenia and/or osteoporosis in older men.

CRF indicative of habitual physical activity has been used as an alternative to examine the association between physical activity and bone health. However, a majority of previous studies examining the relationship between physical activity and bone health have been conducted in women and showed that both resistance and weight-bearing aerobic exercises are significantly associated with increased bone mass and strength [26], attenuated aged-related loss in BMD [16], and decreased risk for bone fracture [27]. Unlike women, however, evidence is not enough to recommend resistance or weight-bearing exercise as a nonpharmacologic strategy for improving or maintaining BMD in older men [28,29]. For example, previous studies examining the effects of exercise training on BMD in men led to conflicting results due to small sample sizes [30]. Other studies were conducted in healthier and younger men with better bones and fewer covariates [31,32]. In this aspect, we believe that the current study is an important addition to the literature, by reporting the relationship between eCRF and bone health in a large sample of older men in models fully adjusted for covariates.

Several explanations can be given for the current study findings. First, the attenuating effect of high eCRF against the risk of low BMD may be attributed to physical activity-induced increase of mechanical loading and stimulation of new bone formation at the stressed skeletal sites. Participation of regular physical activity results in a number of bone health benefits, including increases in BMD and bone size and increases cortical area and strength during adolescence [33] and adulthood [34], as well as reduced risk for bone fracture later in life [35].

Second, higher serum vitamin D in conjunction with lower serum PTH level by eCRF category (from low to high) may explain the decreased risk of low BMD observed among individuals with middle and/or high eCRF levels. Serum vitamin D level is inversely correlated with serum PTH level; Vitamin D insufficiency causes an increase in serum PTH and in mineral release from bone. In the meantime, chronically elevated serum PTH secondary to vitamin D insufficiency may stimulate bone turnover, causing negative bone balance and increased risks for low BMD and bone fracture. We speculate that higher serum vitamin D levels observed in the middle and high eCRF groups compared with the low eCRF group may be due to group differences in body fat [36] or nutritional intake [37] or outdoor activity [38,39]. In particular, the importance of the nutritional intake of vitamins D and B and calcium in relation to bone health was previously reported from recent studies using a nationwide survey data [6,37].

Lastly, the relationship between eCRF and femoral neck BMD may be influenced by several covariates such as genetics, growth factors, gender, age, body composition, health behaviors (i.e., smoking and alcohol intake), medications, hormones, and nutrition [40]. In this study, the association between higher eCRF and lower risk of low BMD was tested in a model fully adjusted for age, SES, WC, smoking, heavy alcohol intake, serum hormones (vitamin D and PTH), and dietary factors (caloric intake, protein, calcium, and vitamins A and C), minimizing the influence of the covariates on the relationship between eCRF and risk of low BMD.

This study has limitations. First, any causal inference on the relationship between eCRF and BMD cannot be possible. Second, a bidirectional relationship between CRF and BMD can be possible. That is, younger men and/or men with better overall health are more likely to participate in regular physical activity and be physically fit and have healthier bone. Third, the cellular and molecular mechanisms by which high CRF decreases the risk for low BMD remain to be elucidated. Lastly, the validity of the eCRF algorithm used in this study should be confirmed in older Korean populations.

Despite the limitations, we believe that the current findings of the study support and extend the literature regarding physical fitness and bone health by reporting the relationship between higher eCRF and lower risk for osteopenia and osteoporosis in a higher risk group of advanced age in models fully adjusted for a number of potential covariates.

## 5. Conclusions

In this cross-sectional study, we examined the relationship between eCRF and BMD in 2715 older Korean adults aged 50 years and older and showed that higher eCRF was significantly associated with attenuated age-related loss in BMD and decreased risk of low BMD. Together, the current findings of the study have some practical and clinical implications. First, maintaining high CRF via regular exercise is important for the maintenance of good bone health and the prevention of osteoporosis and fracture as well as premature death from hip fracture in older Korean men. Second, assessment of eCRF using routinely obtained health measures is highly feasible in clinics and hospitals and should be considered as a diagnostic tool for bone health status.

## Figures and Tables

**Table 1:** Description of measured parameters according to estimated cardiorespiratory fitness (eCRF) levels.

Variables	All (*n* = 2715)	eCRF Levels			*p* for Linear Trends
		Low (*n* = 678)	Middle (*n* = 1351)	High (*n* = 686)	
**Anthropometrics**					
Age (years)	62.5 ± 8.6	70.6 ± 7.6	61.5 ± 7.6	56.4 ± 5.2	<0.001
Body mass index (kg/m^2^)	23.8 ± 2.9	24.2 ± 3.1	23.8 ± 3.0	23.3 ± 2.5	<0.001
Waist circumference (cm)	85.2 ± 8.5	87.5 ± 9.3	85.3 ± 8.5	82.9 ± 7.2	<0.001
Resting heart rate (beats/min)	70 ± 9	72 ± 10	70 ± 9	67 ± 8	<0.001
Heavy alcohol drinkers, *n* (%)	609 (22.4)	232 (38.1)	265 (43.5)	112 (18.4)	<0.001
Current smokers, *n* (%)	1589 (70.2)	366 (23.0)	779 (40.9)	444 (27.9)	<0.001
**Socio-economic Status**					
Education, *n* (%)					<0.001
Elementary	873 (32.3)	317 (36.3)	407 (46.5)	150 (17.2)	
Middle and high schools	1323 (48.7)	271 (20.5)	682 (51.5)	370 (28.0)	
College or higher	519 (19.1)	90 (17.3)	263 (50.7)	166 (32.0)	
Household income (won)	340 ± 1172	210 ± 405	340 ± 778	469 ± 2015	<0.001
**Physical Activity and Fitness**					
Physical activity score	3.36 ± 1.55	1.83 ± 0.54	3.31 ± 1.48	4.95 ± 0.21	<0.001
eCRF (METs)	10.0 ± 1.7	7.9 ± 0.6	10.0 ± 0.9	12.2 ± 0.5	<0.001
**Serum Vitamin and Hormones**					
Vitamin D (ng/mL)	21.5 ± 7.5	21.1 ± 7.7	21.3 ± 7.3	22.1 ± 7.6	0.004
Parathyroid hormone (*p*g/mL)	65.5 ± 26.4	69.1 ± 31.7	65.0 ± 24.6	63.5 ± 24.1	<0.001
**Nutrient intake**					
Energy (kcal/day)	2139 ± 789	1904 ± 655	2166 ± 803	2340 ± 828	<0.001
Protein (g/day)	75 ± 35	64 ± 30	76 ± 36	83 ± 36	<0.001
Calcium (mg/day)	554 ± 330	488 ± 361	575 ± 399	585 ± 330	<0.001
Vitamin A (μgRE)	847 ± 876	706 ± 770	864 ± 811	966 ± 1,074	<0.001
Vitamin C (mg/day)	111 ± 92	94 ± 79	116 ± 99	120 ± 88	<0.001

eCRF: estimated cardiorespiratory fitness; METs: metabolic equivalents. *p* values of less than 0.05 indicate significant linear trends according to incremental eCRF categories (from low to high).

**Table 2:** Femoral neck bone mineral density (BMD) by eCRF category.

Variables	All (*n* = 2715)	eCRF Levels			*p* for Trends
		Low (*n* = 678)	Middle (*n* = 1351)	High (*n* = 686)	
BMD (g/cm^2^)	0.75 ± 0.11	0.71 ± 0.12	0.75 ± 0.12	0.78 ± 0.11	<0.001
T-score (mean ± SD)	−0.76 ± 0.95	−1.06 ± 0.98	−0.74 ± 0.98	−0.51 ± 0.88	<0.001
Osteopenia (*n*, [%])	1044 (38.5)	320 (47.2)	524 (38.8)	200 (29.2)	<0.001
Osteoporosis (*n*, [%])	71 (2.6)	41 (6.0)	25 (1.9)	5 (0.7)	<0.001
Low BMD (*n*, [%])	1115 (41.1)	361 (53.2)	549 (40.6)	205 (29.9)	<0.001
Normal (*n*, [%])	1600 (60.5)	317 (49.8)	802 (60.5)	481 (70.6)	<0.001

eCRF: estimated cardiorespiratory fitness; osteopenia is defined as a T-score of −1.0 or less and greater than −2.5, and osteoporosis is defined as a T-score of −2.5 or less; low BMD includes both osteopenia and osteoporosis; the cut-off eCRF values were ≤7.9 METS for low, 8.0~12.1 METs for middle, and ≥12.2 METs for high groups.

**Table 3:** Odds ratios (OR) and 95% confidence intervals (CI) for osteopenia, osteoporosis, and low bone mineral density by eCRF category.

Variables	Low eCRF	Middle eCRF	*p* Value	High eCRF	*p* Value
**Osteopenia**					
Crude OR (95% CI)	1	0.647 (0.535–0.783)	<0.001	0.412 (0.328–0.517)	<0.001
Adjusted ^a^ OR (95% CI)	1	0.923 (0.703–1.213)	0.565	0.692 (0.480–0.998)	0.049
**Osteoporosis**					
Crude OR (95% CI)	1	0.241 (0.144–0.403)	0.001	0.080 (0.031–0.206)	0.001
Adjusted ^a^ OR (95% CI)	1	0.708 (0.308–1.632)	0.418	0.354 (0.089–1.413)	0.142
**Low Bone Mineral Density**					
Crude OR (95% CI)	1	0.601 (0.499–0.724)	<0.001	0.374 (0.300–0.407)	<0.001
Adjusted ^a^ OR (95% CI)	1	0.905 (0.691–1.184)	0.467	0.669 (0.497–0.966)	0.029

eCRF, estimated cardiorespiratory fitness; BMD, bone mineral density. Osteopenia is defined as a T-score of −1.0 or less and greater than −2.5, and osteoporosis is defined as a T-score of −2.5 or less; low BMD includes both osteopenia and osteoporosis. ^a^ Model adjusted for age, education, household income, waist circumference, heavy alcohol intake, smoking, serum vitamin D, serum parathyroid hormone, caloric intake, protein intake, calcium intake, vitamin A intake, and vitamin C intake.
